# *Bacillus megaterium*: Evaluation of Chemical Nature of Metabolites and Their Antioxidant and Agronomics Properties

**DOI:** 10.3390/ijms25063235

**Published:** 2024-03-12

**Authors:** Anna Hur, Mohamed Marouane Saoudi, Hicham Ferhout, Laila Mzali, Patricia Taillandier, Jalloul Bouajila

**Affiliations:** 1 Laboratoire de Génie Chimique, UMR 5503, Université de Toulouse, CNRS, INPT, UPS, 31062 Toulouse, France; anna.hur@univ-tlse3.fr (A.H.); mohamed.saoudi@univ-tlse3.fr (M.M.S.); patricia.taillandier@ensiacet.fr (P.T.); 2 Agronutrition, Rue Pierre et Marie Curie Immeuble, BIOSTEP, 31670 Labège, France; h.ferhout@agro-nutrition.fr (H.F.); l.mzali@agro-nutrition.fr (L.M.)

**Keywords:** *Bacillus megaterium*, extraction, metabolites, bioactive compounds, germination

## Abstract

*Bacillus megaterium* is particularly known for its abundance in soils and its plant growth promotion. To characterize the metabolites excreted by this specie, we performed successive liquid/liquid extractions from bacteria culture medium with different polarity solvents (cyclohexane, dichloromethane, ethyl acetate and butanol) to separate the metabolites in different polarity groups. The extracts were characterized regarding their total phenolic content, the amount of reducing sugar, the concentration of primary amines and proteins, their chromatographic profile by HPLC-DAD-ELSD and their chemical identification by GC-MS. Among the 75 compounds which are produced by the bacteria, 19 identifications were for the first time found as metabolites of *B. megaterium* and 23 were described for the first time as metabolites in *Bacillus* genus. The different extracts containing *B. megaterium* metabolites showed interesting agronomic activity, with a global inhibition of seed germination rates of soya, sunflower, corn and ray grass, but not of corn, compared to culture medium alone. Our results suggest that *B. megaterium* can produce various metabolites, like butanediol, cyclic dipeptides, fatty acids, and hydrocarbons, with diverse effects and sometimes with opposite effects in order to modulate its response to plant growth and adapt to various environmental effects. These findings provide new insight into bioactive properties of this species for therapeutic uses on plants.

## 1. Introduction

Nowadays, new tools are required for the resolution of a massive need to feed a growing world population. Improving the production and quality of food is currently problematic, remaining too dependent on antibiotics, synthetic fertilizers or pesticides [1]. The intensive use of these compounds has led to the emergence of pathogen resistance and severe negative environmental impacts and has thus become an important issue of public health and environment pollution [2,3,4]. New biological alternatives are urgently needed to counter and reverse the spread of these issues.

Thus, natural organisms, especially microorganisms like bacteria, still remain the richest and biggest source for new biocontrol or antimicrobial agents and have emerged as a promising alternative to chemical compounds [5,6,7]. There is a large body of literature reporting the potential use of rhizosphere-associated bacteria with a positive effect on the growth, development or health of plants [8,9]. Under specific environment conditions, plant growth-promoting rhizobacteria (PGPR) can enhance the productivity of field crops, and some species are actually already commercialized for their direct inoculation in soil [10]. Among them, the *Bacillus* genus is one of the most extensively studied and is among the most beneficial bacteria, being mostly exploited as microbial biopesticides [11].

Several species belonging to the genus *Bacillus* have been reported effective for the biocontrol of multiple plant diseases [12]. These species, by colonizing root systems and the surrounding soil layer (rhizosphere), influence the plant through direct growth stimulation and/or by protecting it from infection by phytopathogens [13,14]. According to their localization, these bacteria can have beneficial protective effect by different mechanisms. The external presence of bacteria, in the surrounding soil layer, can improve soil nutrient availability such as solubilized phosphate, potassium, zinc, calcium and magnesium, or fixed nitrogen [15] and protect the plant against heavy metal toxicity [16]. In addition, while bacteria are internal to the plant root, they mitigate plant stress factors and secrete phytohormones [15]. The protection against pathogens is manifold and is also dependent on the localization of the bacteria. The internal mechanism corresponds to the activation of plant defense system by rhizobacteria-induced systemic resistance (ISR) [12]. External mechanisms correspond to the secretion of antibiotics, the formation of biofilm on root, or the competition for space and nutrients [12,13,16]. However, the modification of the bio-balance of living organisms in soil is not yet well understood. Over the long term, it can have an effect on bio-pollution and can cause an imbalance in some ecosystems [12]. In addition, the direct inoculation of responsible metabolites, like the antibiotics iturin A, surfactin and fengycin, instead of the entire organism has been shown to deliver positive results in several studies [17]. In addition, the production of metabolites from *Bacillus* is already used in industries other than agriculture: medical, chemical, or food industries [18].

In addition to that, some species of *Bacillus* have been studied more extensively than others, probably depending on their discovery date and their abundance in soils. The most described production of *Bacillus* found in the literature relate to *B. subtilis*, *B. thuringiensis*, *B. cereus*, *B. licheniformis*, *B. amyloliquefaciens* and *B. anthracis* [17]. *B. megaterium* production has also been described but to a lesser extent, and the studies on it are more in relation to its utilization as a vector in recombinant protein production [19]. However, *B. megaterium* is known for its abundance in soils, its endophyte colonization of numerous plants and its plant growth promotion [17]. In this regard, *B. megaterium* has already been commercialized for agricultural applications through its inoculation as a living organism [17]. But few *B. megaterium* metabolites have been described so far (phytohormones [20], antibiotic lipopeptides [21], vitamin B12 [22], siderophores [23], biopolymer [18], carotenoids [23], exopolysaccharides [24]) and valuated through their direct utilization for agriculture industry. In the present work, in order to identify its secondary metabolites related to plant growth promotion, a composition and activity analysis of *B. megaterium* filtrated supernatant was achieved through extraction, chemical family quantification, high-performance liquid chromatography (HPLC), gas chromatography–mass spectrometry (GC-MS), antioxidant analysis and seed germination.

## 2. Results

### 2.1. Chemical Family’s Quantification

To attest the quantity of several chemical families in each extract (reducing sugars, polyphenols, proteins and primary amines), spectrophotometric quantifications were performed (by DNS, Folin–Ciocalteu, Lowry and ninhydrin method, respectively), as reported in Table 1 in mg of eq per gram of extract, and in Table 2 in mg of eq per litter of medium. The metabolites produced by *B. megaterium* were analyzed by comparing the chemical composition of BC extracts with those of CS2 extracts (culture medium used as control).

*B. megaterium* decreases the concentration of reducing sugars in the culture medium. As expected, *B. megaterium* used sugars as an initial carbon source for the cells growing and the metabolites synthesis. In Table 1 (Kruskal–Wallis statistic = 26.49, *p* < 0.0002), Dunn’s test confirms the diminution of reducing sugars for all type of extract from CS2 to BC (BuOH: 415.8 to 56.4 mg/g, Dunn’s *p* = 0.0059; Water: 457.6 to 90.5 mg/g, Dunn’s *p* = 0.0391; Raw: 507.7 to 65.3 mg/g, Dunn’s *p* = 0.0006). The method was validated by comparing the initial concentration of dextrose in CS2 medium and this concentration in the CS2 Raw extract. In Table 2, the magnitude order of the reducing sugars quantity in the CS2 Raw extract, representing 15.1 g/L eq, is similar to the initial concentration of dextrose in CS2 medium, 20 g/L.

No conclusion on variation of polyphenols concentration in *B. megaterium* medium can be made. In Table 1 (Kruskal–Wallis statistic = 253.1, *p* < 0.0001), Dunn’s test confirms the increase in polyphenols quantification after the production of *B. megaterium* from CS2 to BC, for the Cyclo extracts (1.7 to 28.7 mg/g, Dunn’s *p* = 0.0488), and for the BuOH extracts (27.7 to 95.6 mg/g, Dunn’s *p* = 0.0188). But a global diminution is observed in raw extracts from CS2 to BC (32.6 to 15.0 mg/g, Dunn’s *p* = 0.0263). However, these slight variations cannot lead to a conclusion on the consumption or production of polyphenols by *B. megaterium*. Indeed, because of the interference of sugars and proteins with the Folin–Ciocalteu method [25], the important quantities of these components in the extracts (Table 1) can lead to a misinterpretation of the variation in polyphenols. This assumption can be confirmed by the comparison of the polyphenol’s concentration in CS2 raw extract, to the initial composition of CS2 medium. Indeed, the polyphenols for the CS2 raw extract represent 969.9 mg/L eq in the initial medium (Table 2). Yet, the unique source of polyphenols can be the yeast extract component (1 g/L) but polyphenols should not represent the entire portion of this component [26].

*B. megaterium* decreases the concentration of proteins in its medium. In Table 1 (Kruskal–Wallis statistic = 21.95, *p* < 0.0005), Dunn’s test confirms the diminution of proteins after the production of *B. megaterium* for the Raw extract from CS2 to BC (219.3 to 165.4 mg/g, Dunn’s *p* = 0.0014) but also for BuOH extract (207.0 to 105.2 mg/g, Dunn’s *p* = 0.0019) and can indicate the consumption of these compounds by *B. megaterium*. The method was validated by comparing the initial concentration of proteins in the CS2 medium and this concentration in the CS2 Raw extract. In Table 2, the proteins for the CS2 Raw extract, representing 6.5 g/L eq, are close to the initial concentration of the peptone and yeast extract in CS2 medium (6 and 1 g/L, respectively).

Primary concentration of amines does not vary in the *B. megaterium* medium. In Table 1 (Kruskal–Wallis statistic = 25.17, *p* < 0.0003), Dunn’s test cannot determine if there is a difference between CS2 and BC for the Raw extracts (18.4 mg/g and 24.5 mg/g, Dunn’s *p* = 0.3941). However, a significant augmentation for the Water extracts (13.5 to 29.5 mg/g, Dunn’s *p* = 0.0075) is observed, but could be influenced by the modifications of the proportion of reducing sugars, polyphenols or proteins of this extract and does not necessarily lead to the production of primary amines in the extract. The method can be validated by comparing the primary concentration of amines in CS2 Raw extract in mg/L (Table 2) to the initial concentration of the peptone and yeast extract. Indeed, the combination of this concentration (547.9 mg/L eq) to the proteins concentrations, is close to the combination of the initial concentration of the peptone and yeast extract (6 and 1 g/L, respectively).

The spectrophotometric quantifications (by Folin–Ciocalteu, DNS, ninhydrin and Lowry method, respectively) for BC extracts of *B. megaterium* culture and CS2 extracts of control medium were obtained. The reference is an equivalent of each family (gallic acid, glucose, glycine, standard proteins, respectively). The results are therefore expressed as an equivalent quantity (mg) in one gram of extract. For each quantification method, the Kruskal–Wallis test is performed. If Kruskal–Wallis test is significant for the assay (*p* < 0.05, represented by a star), Dunn’s test is performed for the 2-by-2 extract rank comparisons. Values in the same column that are labeled with different letters (a–f) differ significantly (*p* < 0.05). Not-analyzed extracts are denoted by na.

The spectrophotometric quantifications (by Folin–Ciocalteu, DNS, ninhydrin and Lowry method, respectively) use an equivalent of each family (gallic acid, glucose, glycine and standard proteins, respectively). The results of mass concentration are expressed as an extraction quantity of extract (mg) for one litter of medium and the chemical quantification are expressed as an equivalent quantity (mg) in one litter of initial liquid medium (corresponding to the combination of results of Table 1 to the mass of extract in one litter of medium). No repetition for the determination of mass concentration was performed, which obstructs the use of statistical tests.

### 2.2. Chemical Identification by GC-MS

In order to further elucidate the active compounds produced by *B. megaterium*, the different extracts of the inoculated medium (BC extracts) were subjected to GC-MS analysis and compared with the extracts of control medium (CS2 extracts).

A total of 75 compounds were found to be present in BC extracts and not present in CS2 extracts, as illustrated in Table 3 for analysis without derivatization, and in Table 4 for analysis with derivatization. Some of these identified compounds were strictly found in BC extracts, suggesting a probable production by *B. megaterium*: 10 fatty acids derivatives (23′, 32′, 34′, 39′, 42′, 44′, 46′, 47′, 36, 44), 5 linear hydrocarbons (4, 12, 20, 24, 25), 5 cyclic hydrocarbons with 3 isomers (5, 6, 8, 9, 10, 11, 15, 18), 6 amino acids (10′, 12′, 15′, 18′, 20′, 22′), 2 dipeptides (29, 47), 2 cyclic dipeptides (46, 64), 6 aromatic hydrocarbons (1, 16, 26, 27, 31, 33), 2 phthalates (59, 61), 2 sugar acids with 1 isomer (9′, 24′, 41′), 4 polyols with 1 isomer (2′, 4′, 5′, 6′, 21′), 2 cyclitols (29′, 43′) and 17 others were identified (3, 7, 13, 14, 19, 21, 23, 32, 37, 43, 45, 48, 50, 62, 63, 17′, 38′).

### 2.3. HPLC Analysis

**Figure 1:**
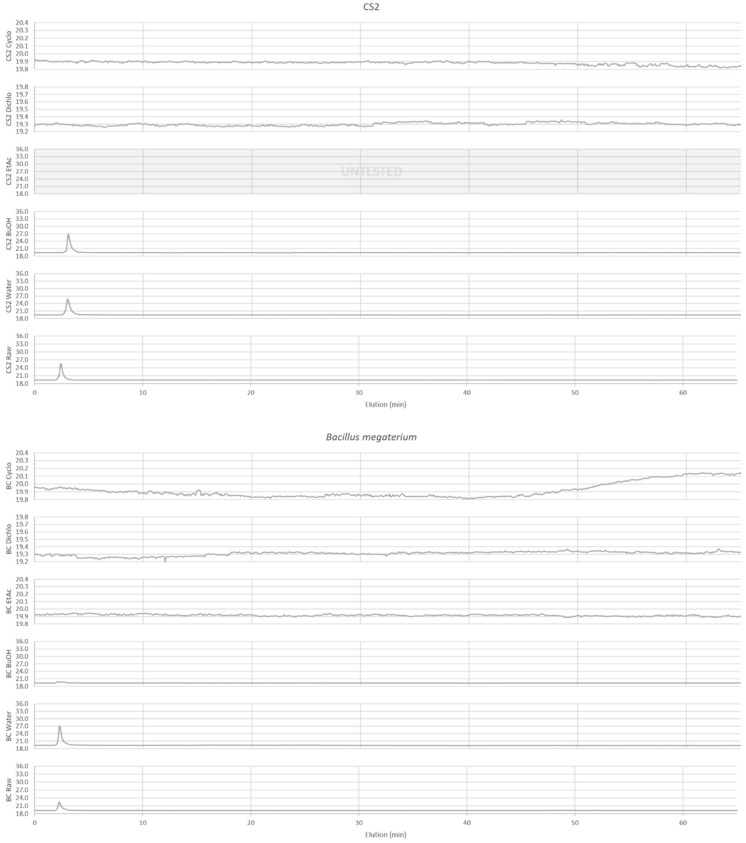
HPLC-ELSD acquisition for BC extracts of *B. megaterium* culture and CS2 extracts of control medium. Apolar (Cyclo, Dichlo and EtAc) extracts are injected at 2 mg/mL and polar extracts (BuOH, Water, Raw) at 20 mg/mL.

**Figure 2:**
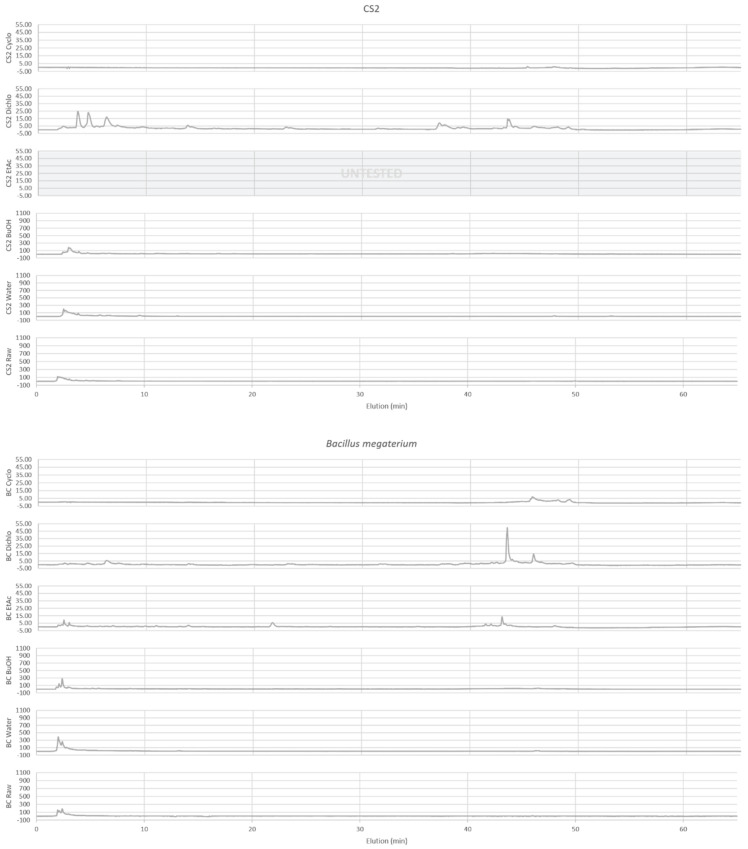
HPLC-DAD acquisition for BC extracts of *B. megaterium* culture and CS2 extracts of control medium. Apolar (Cyclo, Dichlo and EtAc) extracts are injected at 2 mg/mL and polar extracts (BuOH, Water, Raw) at 20 mg/mL.

The extracts analysis by HPLC facilitated the definition of a chemical profile whose chromatograms are visible in Figure 1 and Figure 2.

Sugars are apparent with ELSD in polar extracts. ELSD chromatograms (Figure 1) of polar extracts (BuOH, Water, Raw) for CS2 and BC, show the presence of compounds with high polarity (t = 2.109–3.129 min), which could correspond to sugars observed with the chemical quantification described above (Table 1), and GC-MS (Table 4: 25′, 26′, 27′, 28′, 30′, 31′, 33′, 35′, 36′, 37′, 40′, 45′, 48′). A decrease in these compounds is observed in BC BuOH and Raw extracts compared to CS2 extracts, confirming the consumption of sugars by *B. megaterium* observed with our previous quantifications. But this variation is not seen for BC Water extract with ELSD chromatograms contrary to what we had previously described with the spectrophotometric quantification in this extract. No peak is detected on the chromatogram of evaluated apolar extracts (BC Cyclo, BC Dichlo, BC EtAc, CS2 Cyclo, CS2 Dichlo), confirming the absence of sugars in these extracts and in accordance with our previous results with GC-MS (Table 4). However, this absence of peak means that main compounds detected with GC-MS in these extracts are not apparent with ELSD, like cyclic dipeptides (Table 3: 28, 30, 34, 35, 38, 39, 40, 41, 46, 49, 51, 54, 57, 64), polyols (Table 4: 4′, 5′, 6′, 13′) and fatty acids (Table 4: 23′, 32′, 34′, 39′, 42′, 44′, 46′, 47′). These results can show that principal compounds cannot be detected by ELSD, either because they are not volatile enough, their concentrations are under the detection limit of 50 mg/L, or their main compounds are not soluble in 20/80 acetonitrile/water.

Polyphenols, aromatic proteins and aromatic cyclic dipeptides are apparent with DAD. DAD chromatograms (Figure 2) of polar extracts for CS2 and BC, show several peaks from 2 min for the most intense and spreading until 20 min for the least intense. Polyphenols, quantified in CS2 and BC extracts as described above (Table 1) and including several phenols found in polar extracts by GC-MS (Table 3: 53, 55, 56, 58), are a family well known to be detected at 280 nm. Proteins detected in polar extracts (Table 1) could correspond to hydrophilic proteins with a relative presence of aromatic amino acids, that can also be detected at 280 nm. For the apolar extracts, the DAD chromatogram shows several peaks (except for the Cyclo extracts) and with different elution times between extracts, indicating different compounds for each extract. In addition BC Dichlo chromatogram exhibit only peak with important elution time (t = 43.40–45.88 min), the others (CS2 Dichlo and BC EtAc) present peaks, spreading from 1.872 to 49.054 min. Polyphenols, also quantified in CS2 and BC apolar extracts (Table 1) and detected by GC-MS (Table 3: 21, 22, 37, 43, 53, 55, 56, 58), compounds with aromatic amino acids detected by GC-MS (Table 3: 46, 47, 49, 51, 54, 57; Table 4: 22′), and other aromatics detected by GC-MS (Table 3: 1, 7, 13, 14, 16, 23, 26, 27, 31, 32, 33, 42, 48, 50, 59, 60, 61; Table 4: 17′) should correspond to these peaks.

### 2.4. Antioxidant Activity (DPPH)

**Figure 3:**
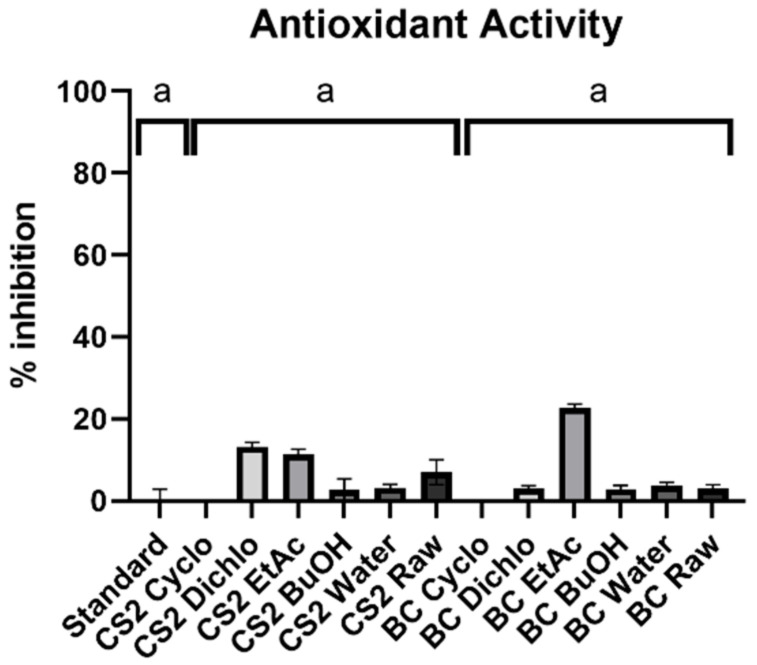
Antioxidant activity by the DPPH method, for BC extracts of *B. megaterium* culture and CS2 extracts of control medium. The results are therefore expressed as a percentage of inhibition of DPPH free radical. If the Kruskal–Wallis test is not significant (*p* > 0.05), groups are labeled with the same letters (a).

The antioxidant activity of each extract was determined by DPPH method, allowing us to quantify the inhibition of a free radical, as shown in Figure 3. In order to elucidate if some compounds produced by *B. megaterium* present an antioxidant activity, the analysis between grouped BC extracts and grouped CS2 extracts was performed. The comparison of these two groups with the standard (DPPH test for the solvent of samples) is also performed.

The antioxidant activity of compounds in the supernatant do not significantly vary after *B. megaterium* culture. The Kruskal–Wallis (statistic = 5.29, *p* < 0.0652) test cannot determine if there is a difference between CS2 extracts, BC extracts, and the standard. Thus, no global difference is observed between BC extracts and CS2 extracts, regarding the antioxidant activity.

### 2.5. Agronomic Activity (Corn, Sunflower, Soya and Ray Grass)

**Figure 4:**
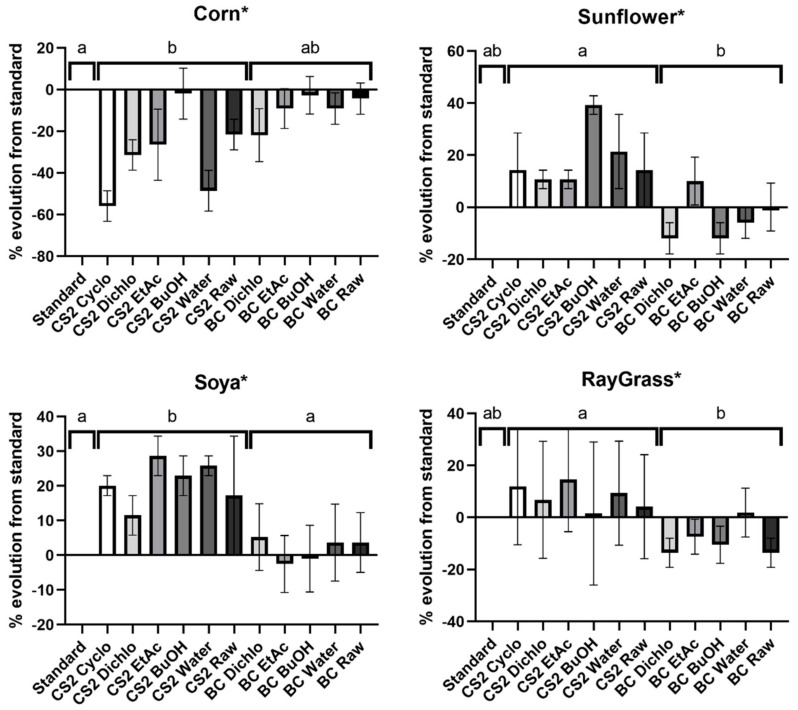
Agronomic activity on corn, sunflower, soya and ray grass, for BC extracts of *B. megaterium* culture and CS2 extracts of control medium. The results are therefore expressed as a percentage of evolution compared to standard gemination. For each seed, a Kruskal–Wallis test is performed. If the correlation is significant for the assay (*p* < 0.05, represented by a star), Dunn’s test is performed for the 2-by-2 extract rank comparisons: values that are labeled with different letters (a and b) differ significantly (*p* < 0.05).

The agronomic activity of extracts is evaluated by applying them on seeds and analyzing the augmentation or the inhibition of the germination rate compared to standard germination, as shown in Figure 4. For the control with water, the number of seeds that germinated at the maximum duration is 21 for corn, 17 for sunflower, 21 for soya and 21 for ray grass. The analysis between grouped BC extracts and grouped CS2 extracts was performed. The Kruskal–Wallis test was significant for all seed germination: corn (Kruskal–Wallis statistic = 6.709, *p* = 0.0164), sunflower (Kruskal–Wallis statistic = 9.147, *p* = 0.0015), soya (Kruskal–Wallis statistic = 9.096, *p* = 0.0015) and ray grass (Kruskal–Wallis statistic = 8.550, *p* = 0.0032).

*B. megaterium* metabolites are inefficient on seed germination compared to the standard, but they seem to inhibit it compared to the culture medium CS2 alone. BC extracts do not present an obvious activity and seem quite inefficient: BC extracts is not significantly different from the standard for corn (Dunn’s *p* = 0.1481), sunflower (Dunn’s *p* = 0.8290), soya (Dunn’s *p* = 0.8294) and ray grass (Dunn’s *p* = 0.5587). However, a positive effect of CS2 medium on germination, excepted for corn, is observed. This beneficial activity of the CS2 extracts is quite apparent for three seeds: sunflower (Dunn’s *p* = 0.0578), soya (Dunn’s *p* = 0.0273) and ray grass (Dunn’s *p* = 0.1275). And then, when the activity of BC extracts is compared with CS2 extracts, a significant negative effect of BC extracts is observed on seed germination: BC extracts activity is lower for sunflower (Dunn’s *p* = 0.0043), soya (Dunn’s *p* = 0.0074) and ray grass (Dunn’s *p* = 0.5587).

### 2.6. Principal Components Analysis

**Figure 5:**
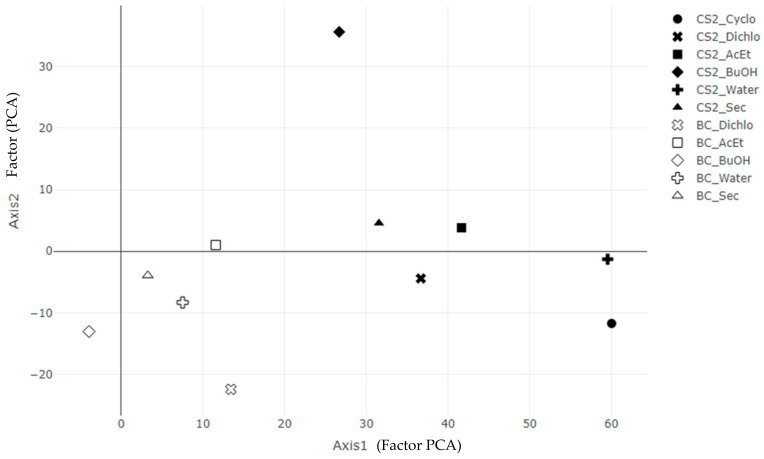
Principal component analysis with the 5 activity variables (germination rates and antioxidant activities) for BC extracts of *B. megaterium* culture and CS2 extracts of control medium. The data are not normalized to include the magnitude of each parameter. The horizontal axis (76.0% of the data variance) corresponds to the following linear combination: 0.140 (antioxidant) − 0.786 (corn) + 0.359 (sunflower) + 0.454 (soya) + 0.166 (ray grass). The vertical axis (13.7% of the data variance) corresponds to the following linear combination: −0.002 (antioxidant) + 0.538 (corn) + 0.786 (sunflower) + 0.241 (soya) + 0.188 (ray grass).

The principal component analysis (PCA), shown in Figure 5, is performed with the 5 activity variables (germination rates and antioxidant activities), to complete a comparison of global activity between each extract. As the data are not normalized, this PCA aims at including the magnitude of each parameter. The horizontal axis represents 76.0% of the data variance, while the vertical axis represents 13.7% of the variance. Thus, the PCA displays almost 90% of the global information. The horizontal axis is relative to the germination increase in sunflower, soya and ray grass, and to the inhibition of corn and DPPH. The vertical axis is relative to the germination increase in sunflower, soya, ray grass and corn (antioxidant activity is negligible).

The inhibition of germination by *B. megaterium* metabolites: Unlike CS2 extracts, which have a global increasing effect on the germination rate (except for corn) (axis 1), BC extracts present a global inhibition effect on all seeds but with a smaller magnitude (axis 2).

## 3. Discussion

Little documentation exists about metabolite production of *B. megaterium*. To determine the nature of these compounds, quantitative and qualitative analyses have been performed. The quantification of sugars, polyphenols, proteins and primary amines, as well as the HPLC analysis and GC-MS analysis, have allowed us to determine their repartition, their nature and their transformation in different extracts. Thus, the main components of the initial culture medium, as sugars and proteins, have been mostly consumed by *B. megaterium*, as seen by chemical family quantification, GC-MS (25′, 26′, 27′, 28′, 30′, 31′, 33′, 35′, 36′, 37′, 40′, 45′, 48′) and ELSD in some polar extracts.

However, the production of other proteins and the polymerization of sugars by *B. megaterium* can be suspected. Indeed, the none-diminution in the sugar peak intensity on ELSD chromatograms in the Water extract could suggest the presence of exopolysaccharides. The fact that sugar reducing function in polysaccharides is not available for the DNS reaction [27] and that these compounds are not volatiles could explain why a diminution in sugars is seen via quantification and why it is not detected by GC-MS. These results are supported in the literature by the description of some exopolysaccharides secretions by *B. megaterium* [24]. Several studies have also described *B. megaterium* as a producer of extracellular proteins [19,28]. It could explain that proportion of proteins is still important in BC polar extracts with 10.5 to 19.4%. It could also explain that intensity and nature of cyclic dipeptides are changing from CS2 to BC (28, 30, 34, 35, 38, 39, 40, 41, 46, 49, 51, 54, 57, 64) and that different peaks on DAD chromatogram suggesting different aromatic compounds from CS2.

A GC-MS analysis of the BC Cyclo extract allow us to highlight the presence of fatty acids (23, 32, 34, 39, 42, 44, 46, 47) and fatty aldehyde (36′, 44′). Despite the absence of a conclusion on the polyphenol production by *B. megaterium* through the Folin–Ciocalteu method, polyphenol profiles by GC-MS evolved after *B. megaterium* production (21, 22, 37, 43, 53, 58). These results confirm previous internals results which showed that *B. megaterium* produces several phenols [29]. A GC-MS analysis of BC Dichlo highlights the presence of cyclic dipeptides (28, 34, 35, 38, 39, 40, 41, 46, 49, 54, 57, 64) and butanediol (5′, 6′). Less information is available for BC EtAc and BuOH extracts, with the description of butanediol (5′, 6′) mostly being available.

However, this work is the first description of numerous compounds as *B. megaterium* metabolites: tridecanoic acid (23′), myristoleic acid (34′), methyl palmitate (C16:0) (36), undecane (4), dodecane (12), heptadecane (25), erucamide (62), squalene (63), cyclo(phe-phe) (64), cyclo(ala-phe) (46), pseudocumene (1), m-di-tert-butylbenzene (16), ethyl 4-ethoxybenzoate (23), 2,4-dimethylbenzaldehyde (14), ribonic acid (24′, 41′), ethylene glycol (2′) and propylene glycol (4′), 2,3-butanediol (5′, 6′) and L-threitol (21′). The description of 2,3-butanediol (5′, 6′) has already been provided in other laboratory work [30]. For other compounds, it is even the first description of metabolites in the *Bacillus* genus: methyl iso-stearate (iC18:0) (44), 2-methyl-trans-decalin (5, 6, 8), 2-methyl-cis-decalin (9, 10), 2,6-dimethyldecalin (11), hexylcyclohexane (15), 1,1′-bicyclohexyl (18), 9,17-octadecadienal (45), phenyl-alkanes (26, 27, 31, 33), di-2-propylpentyl-phthalate (59) and the di-2-ethylhexyl-isophthalate (61), di-t-butyl-1-oxaspiro(4,5)deca-6,9-diene-2,8-dione (37), 2,4,6-triisopropylphenol (43), 3,4-dimethylbenzamide (19), octinoxate (50) and dihydroisophorone (3), tert-octyldiphenylamine (48), n-butylbenzenesulfonamide (32), myo-inositol (43′) and D-pinitol (29′), and D-galactose oxime (38′). These metabolites identified for the first time provide a wealth of chemical families for varied applications.

Correlating the nature of each extract to their activity allows us to better understand the chemical mechanisms of *B. megaterium* effect on plants. It seems that the compounds produced by *B. megaterium* do not have an enough antioxidant activity to be detected. In fact, the antioxidant activity of DPPH is usually highly related to the presence of polyphenols in samples [31], thus supporting results on the polyphenol production by *Bacillus*.

On the other hand, agronomic results indicate that some extracts containing metabolites of *B. megaterium* showed interesting biological activities. Most of the extracts of *B. megaterium* seem to inhibit seed germination compared to initial medium extracts and compared to standard. This inhibition of seed germination is surprising given that it is widely known in the literature that *Bacillus* species are a predominant plant growth-promoting bacterium. In addition, compounds of initial medium in BC extracts also present plant growth benefices, like some cyclic dipeptides [32]. However, internal works in the laboratory have been previously conducted, describing the effect of *B. megaterium* medium extraction on maize and sunflower, and showing either an inhibition or an increase in germination [30]. The inhibition of seed germination by BC extracts could be explained by the present of several compounds involved in plant defense that negatively affect the plant growth to deviate their fundamental functions to ensure this purpose. Indeed, dodecane (12) and undecane (4) are elicitors of ISR [33], and the latter even seems to decrease plant biomass [34]. Despite the capacity of erucamide (63) to improve nitrogen metabolism, this compound is mainly produced when the plant is under important stress [35] and its production seems also to be correlated with a decrease in plant growth [36]. In addition, the 2,4-Di-tert-butylphenol (22) can induce systemic resistance against pathogenic fungi [37] and at high concentrations, it also limits plant growth [38]. In addition, 2,6-di-tert-butyl-P-benzoquinone (21) is part of p-benzoquinones, known to inhibit root development and elongation [39]. In the same way, 2,3-butanediol (5′, 6′), identified by GC-MS with derivatization in BC Dichlo, AcEt, BuOH and Water, seems to have a particular importance for defense in some plants and has been confirmed to be necessary for activating the ISR process [40,41]. The L-threitol (21′) seems involved in pathogens signaling [42,43] and 7,9-di-tert-butyl-1-oxaspiro (4,5) deca-6,9-diene-2,8-dione (37) is present in plants which are able to defend against parasites [44].

Thus, some compounds seem to deviate plant essential functions for enhance defense, resulting in diminution of plant growth. In addition, most of the research implies the presence of *Bacillus* directly in contact with the plant rhizosphere and can indicate that these beneficial effects require the presence of the bacterium during plant growth and explain the different results of its direct use as an inoculant in the field [45]. In addition, the main compounds of BC extracts have not been determined and some are suspected, like proteins, exopolysaccharides and polyphenols. In fact, the presence of numerous other compounds detected by GC-MS that could not be identified with the current library highlights the potential of *B. megaterium* to produce new unidentified compounds. Thus, further investigations are necessary to separate these compounds and to provide more accurate correlations between the activity and the nature of each compound.

This work helps us to better understand the nature of the metabolites of *B. megaterium* and their effects, and it is the first step to understand the mechanisms of action of this bacterium as a PGPR and finally value specific and restricted compounds, in order to simplify but mostly control the uses of agronomic products.

## 4. Materials and Methods

### 4.1. Strain and Culture Condition

*B. megaterium* has been isolated from agricultural soils used for barley and wheat and cultivated by Agronutrition (Agronutrition, Labège, France). A sequencing of the bacterial 16S rRNA gene after Polymerase Chain Reaction (PCR) amplification using the universal bacterial primers 27F and 1492R was then performed. To confirm the identity of the bacterium, a comparison was made between resulting sequences and the identity of *B. megaterium* (AGN01; reference Anses (France) Bc07-Bmeg (CECT9639)) in the BLASTn (nucleotides database by NCBI). *B. megaterium* was then cultivated in a liquid CS2 medium (peptone from soybean 6 g/L; yeast extract 1 g/L; dextrose 20 g/L; iron sulphate 0.05 g/L; manganese sulphate 0.05 g/L; antifoaming emulsion 0.28 mL/L; pH 7) at 30 °C during 24 h under agitation. The bacterial culture was centrifuged at 10^4^× *g* for 20 min at 4 °C and filtered through a 0.22 µm filter, to obtain cell-free culture supernatant.

### 4.2. Extraction

The cell-free supernatant of *B. megaterium* culture (BC) was fractioned by successive liquid–liquid extractions [30]. Four solvents with different polarities were selected and used in the order of increasing polarity: cyclohexane (BC Cyclo), dichloromethane (BC Dichlo), ethyl acetate (BC EtAc) and butanol extracts (BC BuOH). The extractions were performed at room temperature with 1 L of solvent for 1 L of medium (1:1). Each organic phase and the residual water (BC Water) were then collected and dried by rotary evaporator at 35 °C (Heidolph, Schwabach, Germany) to generate the extracts. Distinctively, the culture filtrates supernatant without extraction was also dried to prepare another extract (BC Raw). The medium without bacteria culture (CS2 medium) was extracted by the same way, to generate control extracts (CS2 Cyclo, CS2 Dichlo, CS2 EtAc, CS2 BuOH, CS2 Water and CS2 Raw).

### 4.3. Reducing Sugars Quantification

The quantification of reducing sugars amount was performed by the 3,5-dinitrosalicylic acid (DNS) method [46]. The samples were prepared by solubilizing 5 mg/mL of extracts in dimethyl sulfoxide (DMSO) and then diluted at 2 mg/mL to fit the standard range concentrations. Each sample (150 µL) was added to 150 µL of prepared DNS solution (NaOH 2 M, Na_2_CO_3_ 1.8 M, DNS 0.12 M). The mixture was incubated for 5 min at 100 °C and then cooled in ice to stop the reaction. After an addition of 750 µL of water, the absorption was read at 540 nm. The blank was performed by the subtraction of solvents absorption, reagent absorption and sample absorption. The results were expressed as mg of glucose equivalents (eq) per gram of extract (Table 1) and as mg of glucose eq per litter of supernatant (Table 2). Four repetitions were performed for each sample.

### 4.4. Polyphenols Quantification

The quantification of polyphenols amount was performed by the Folin–Ciocalteu method [47]. The samples were prepared by solubilizing 5 mg/mL of extracts in DMSO. Each sample (20 µL) was added to 100 µL of prepared Folin–Ciocalteu solution (0.2 N). The mixture equilibrated with an incubation of 5 min at room temperature and then mixed with 80 µL of 75 g/L sodium carbonate solution. After an incubation of 15 min at room temperature, the absorption was read at 765 nm. The blank was performed by the subtraction of solvents absorption, reagent absorption and sample absorption. The results were expressed as mg of gallic acid eq per gram of extract (Table 1) and then as mg of gallic acid eq per litter of supernatant (Table 2). Four repetitions were performed for each sample.

### 4.5. Proteins Quantification

The quantification of proteins amount was performed by using the Total Protein Kit TP0300 (Micro Lowry, Peterson’s Modification) of Sigma Aldrich (St. Louis, MO, USA) [48]. The samples were prepared by solubilizing 5 mg/mL of extracts in DMSO and then diluted at 0.25 mg/mL to fit in the standard range concentrations. Each sample (80 µL) was added to 80 µL of prepared Lowry Reagent solution. The mixture was incubated for 20 min at room temperature. After an addition of 40 µL of prepared Folin–Ciocalteu solution, the mixture was incubated for 30 min at room temperature. The blank was performed by the subtraction of solvent absorption, reagent absorption and sample absorption. The results were expressed as mg of Protein Standard per gram of extract (Table 1) and as mg of Protein Standard per litter of supernatant (Table 2). Four repetitions were performed for each sample.

### 4.6. Primary Amines Quantification

The quantification of primary amines amount was performed by the ninhydrin method [49]. The samples were prepared by solubilizing 5 mg/mL of extracts in 10% DMSO and then diluted at 0.5 mg/mL to fit in the standard range concentrations. Each sample (240 µL) was added to 120 µL of prepared ninhydrin solution (Na_2_HPO_4_ 0.28 M, NaH_2_PO_4_ 0.44 M, fructose 0.17 M, ninhydrin 0.28 M, pH = 6.7). The mixture was incubated for 15 min at 100 °C and then incubated at room temperature for 10 min. After an addition of 600 µL of water, the absorption was read at 570 nm. The blank was performed by the subtraction of solvents absorption, reagent absorption and sample absorption. The results were expressed as mg of glycine eq per gram of extract (Table 1) and as mg of glycine eq per litter of supernatant (Table 2). Four repetitions were performed for each sample.

### 4.7. HPLC Analysis (HPLC-DAD/ELSD)

The extracts analysis by HPLC (Figure 1 and Figure 2) allows the definition of a chemical profile including the detection of aromatic compounds by DAD (diode array detector) from 200 to 500 nm and the detection of main compounds through ELSD (evaporative light scattering detector) [47]. The wavelength was selected at 280 nm for the visualization of DAD detection. The analysis equipment comprised a liquid chromatography system equipped with an autosampler (SpectraSYSTEM AS3000 (San Jose, CA, USA)), a pump (Dionex P680 HPLC Pump (San Jose, CA, USA)), a degasser (ERMA ERC-3114 (San Jose, CA, USA)), a DAD detector (Waters 996 PDA Detector (Milford, MA, USA)), an ELSD detector (Teledyne Isco 340CF ELSD (Lincoln, NE, USA)) and Chromeleon software 6.8 (Thermofisher, Illkirch-Graffenstaden, France). The stationary phase was a RP-C18 column (Phenomenex, Le Pecq, France), 25 cm × 4.6 mm with 5 μm particle size. The mobile phase was a solvent gradient at a flow rate of 1.2 mL/min, carried out by the variation of solvent A (water pH = 2.65) and solvent B (80/20 acetonitrile/water pH = 2.65). The elution method is as follows: 0–35 min, 12–30% B; 35–40 min, 30–50% B; 40–45 min, 50–100% B; 45–60 min, 100–12% B; 60–65 min, 12% B. The samples were prepared by solubilizing the extracts in 20/80 acetonitrile/water and injected at 20 μL. The Cyclo, Dichlo and EtAc extracts were analyzed at 2 mg/mL and the BuOH, Water and Raw extracts were analyzed at 20 mg/mL.

### 4.8. Chemical Composition (GC-MS and Derivatization Method)

The identification of chemical compounds in the extracts was performed by GC-MS analysis on non-derivatized and derivatized samples [47]. The derivatized samples were prepared by solubilizing 5 mg/mL of extracts in 1 mL of acetonitrile and 0.15 mL of BSTFA (N,O-Bistrimethylsilyltrifluoroacetamide) with 1% TMCS (chlorotrimethylsilane). The humidity was removed by nitrogen circulation on solution for 20 s and the sample were then incubated at 40 °C for 15 min. The non-derivatized samples were prepared by solubilizing 5 mg/mL of extracts in their solvents of extraction, except for Water extracts, solubilized in methanol. The analysis equipment comprised a gas chromatography system (Varian CP-3800). The chromatographic column used was a silica capillary DB-5MS column (5% phenylmethylpolysiloxane, 30 × 0.25 mm, film thickness 0.25 μm), in constant flow mode at 1 mL/min. The samples were injected at 2 μL. The temperature gradient of the method was as follows: 0–5 min, 60 °C; 5–19 min, 60–270 °C; 19–25 min, 270 °C; 25–25.5 min, 270–300 °C; 25.5–30 min, 300 °C. The system was coupled to a mass spectrometer (Varian Saturn 2000 (Le Plessis-Robinson, France), operating with an electron ionization source and an ion trap analyzer. The trap temperature was 250 °C and that of the transfer line was 270 °C. Mass scanning was performed from 40 to 650 *m*/*z*. The processing software was Xcalibur Qual Browser (Thermofisher, Illkirch-Graffenstaden, France). The commercial mass spectra database NIST08 was used for the chemical identification. The identified compounds are visible in Table 3 and Table 4.

### 4.9. Antioxidant Activity

The determination of antioxidant activity of extracts was performed by a chemical method with DPPH (2,2-diphenyl-1-picrylhydrazyl) [47]. The samples were prepared by solubilizing 0.5 mg/mL of extracts in DMSO. Each sample (20 µL) was added to 180 µL of prepared DPPH solution (DPPH 0.2 mM in methanol). After an incubation for 25 min at room temperature, the absorption was read at 524 nm. The blank was performed by the subtraction of solvents absorption and sample absorption. This result was linked to the absorption of DPPH solution to express a percentage of DPPH inhibition (Figure 3). Four repetitions were performed for each sample.

### 4.10. Agronomic Activity (Corn, Sunflower, Soya and Ray Grass)

The agronomic activity was determined by measuring the impact on germination rate. The seeds were sterilized with bleach for 5 min. The samples were prepared by solubilizing 0.25 mg/mL of extracts in 5% DMSO [30]. Each extract (5 µL) was applied on 24 seeds of corn, sunflower, soya or ray grass. A standard was operated by applying the same quantity of water on seeds. The culture was carried out in a specific chamber for the study of in vitro germination (80% humidity and at a temperature of 22 °C). For each batch, 24 seeds were used. The germinating seed was measured over time at 4 days for corn, 6 days for sunflower, 5 days for soya and 10 days for ray grass. The results were expressed in percentage of evolution of germinated seed from standard (Figure 4). Two repetitions were performed for CS2 extracts and three for BC extracts.

### 4.11. Statistical Analysis

Several statistical tests were performed for the results of chemical family quantification and the activity results. To determine the relevancy of results comparison, significance tests were chosen considering the number of repetitions and the distribution models of the measured variables. Thus, Kruskal–Wallis and Dunn’s nonparametric tests were performed with Prism GraphPad 8.2.1 software [50]. The Kruskal–Wallis indicator allows us to determine if there are one or more results deviating from the rank average of all results. If the Kruskal–Wallis test is significant for the assay (*p* < 0.05), Dunn’s test is performed for the 2-by-2 extract rank comparisons. If not, the results are considered as not significantly different.

For each chemical family quantification, Kruskal–Wallis and Dunn’s tests are used to compare the extracts individually, in order to evaluate the evolution of the chemicals repartition.

For each activity, Kruskal–Wallis and Dunn’s tests are used to compare the extracts by groups, in order to evaluate the global evolution of the activity.

In addition to appreciate the global efficiency of the extracts, a principal component analysis was performed by R coding, using ade4 and plotly libraries (Figure 5).

## 5. Conclusions

In conclusion, our in-depth study of the secondary metabolites of *B. megaterium* and their impact on plant growth reveals significant consumption of components from the initial medium, suggesting the potential production of exopolysaccharides and cyclic dipeptides. Furthermore, the extracts obtained are rich in fatty acids, fatty aldehydes, cyclic dipeptides and butanediol.

Although *B. megaterium* compounds show no detectable antioxidant activity, there are some fascinating biological activities, including a surprising inhibition of seed germination. A number of derivatives of these compounds can also inhibit essential plant functions, leading to reduced growth. It is essential to keep the bacteria continuously present during plant growth if these beneficial effects are to be observed.

This study highlights the potential of *B. megaterium* to generate new compounds, opening up new prospects for its bioactive properties in agriculture. It also suggests a therapeutic potential by influencing seed germination and producing a variety of metabolites. With these results, there are many promising applications for this bacterium in agronomic approaches that aim to improve plant growth, and further research is needed to identify precisely which compounds are responsible for these effects.

## Figures and Tables

**Table 1:** Chemical family quantification (polyphenols, reducing sugars, primary amines and proteins).

Medium	Extract	Polyphenols (mg/g) *	Reducing Sugars (mg/g) *	Primary Amines (mg/g) *	Proteins (mg/g) *
CS2	Cyclo	1.7 ± 0.3 ^a^	na	0.1 ± 0.5 ^a^	na
	Dichlo	82.2 ± 2.4 ^bcd^	na	na	na
	EtAc	82.3 ± 1.6 ^bcd^	384.1 ± 5.4 ^abc^	na	na
	BuOH	27.7 ± 1.7 ^abef^	415.8 ± 2.9 ^abd^	18.1 ± 0.6 ^bc^	207.0 ± 3.2 ^ab^
	Water	30.2 ± 1.4 ^bcef^	457.6 ± 3.0 ^ad^	13.5 ± 1.8 ^ab^	202.5 ± 3.4 ^abc^
	Raw	32.6 ± 1.7 ^bcde^	507.7 ± 13.2 ^d^	18.4 ± 5.3 ^bc^	219.3 ± 4.6 ^a^
BC	Cyclo	28.7 ± 4.0 ^bef^	na	na	na
	Dichlo	125.8 ± 8.7 ^d^	na	na	na
	EtAc	25.5 ± 4.2 ^abef^	na	na	na
	BuOH	95.6 ± 2.4 ^cd^	56.4 ± 2.5 ^e^	7.4 ± 0.7 ^ab^	105.2 ± 3.0 ^d^
	Water	16.6 ± 0.8 ^aef^	90.5 ± 2.6 ^bce^	29.5 ± 2.1 ^c^	193.5 ± 4.8 ^bcd^
	Raw	15.0 ± 1.1 ^a^	65.3 ± 1.7 ^ce^	24.5 ± 1.2 ^c^	165.4 ± 4.8 ^cd^

na: not analyzed. Letters a–f mean both within rows and columns with different superscript letters are significantly different (*p* < 0.05). * mg/g dry extract.

**Table 2:** Mass of extracts and chemical family quantification (polyphenols, reducing sugars, primary amines and proteins) in one litter of initial liquid medium for BC extracts of *B. megaterium* culture and CS2 extracts of control medium.

Medium	Extract	Mass Concentration (mg/L)	Polyphenols (mg/L)	Reducing Sugars (g/L)	Primary Amines (mg/L)	Proteins (g/L)
CS2	Cyclo	22.7	0.0 ± 0.0	na	0.0 ± 0.0	na
	Dichlo	58.3	4.8 ± 0.1	na	na	na
	EtAc	154.8	12.7 ± 0.3	0.1 ± 0.0	na	na
	BuOH	3203.6	88.8 ± 5.3	1.3 ± 0.0	57.9 ± 1.9	0.7 ± 0.0
	Water	26,733.3	808.7 ± 37.0	12.2 ± 0.1	361.0 ± 47.9	5.4 ± 0.1
	Raw	29,786.7	969.9 ± 52.0	15.1 ± 0.4	547.9 ± 158.7	6.5 ± 0.1
BC	Cyclo	8.2	0.2 ± 0.0	na	na	na
	Dichlo	128.4	16.1 ± 1.1	na	na	na
	EtAc	872.0	22.2 ± 3.7	na	na	na
	BuOH	3221.9	308.0 ± 7.6	0.2 ± 0.0	23.9 ± 2.1	0.3 ± 0.0
	Water	9795.6	162.6 ± 8.1	0.9 ± 0.0	288.6 ± 20.5	1.9 ± 0.0
	Raw	13,725.5	205.9 ± 14.7	0.9 ± 0.0	336.2 ± 16.9	2.3 ± 0.1

na: not analyzed.

**Table 3:** GC-MS analysis (area ×10^6^) without derivatization for BC extracts of *B. megaterium* culture and CS2 extracts of control medium.

N°	RT (min)	Compound	Formula	Structure	CS2						BC					
					Cyclo	Dichlo	EtAc	BuOH	Water	Raw	Cyclo	Dichlo	EtAc	BuOH	Water	Raw
1	7.56	Pseudocumene	C_9_H_12_	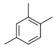	ND	ND	ND	ND	ND	ND	9.2	ND	ND	ND	ND	ND
2	8.14	Eucalyptol	C_10_H_18_O	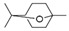	2.9	ND	ND	ND	ND	ND	ND	ND	ND	ND	ND	ND
3	8.43	3,3,5-Trimethylcyclohexanone	C_9_H_16_O	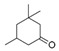	ND	ND	ND	ND	ND	ND	ND	1.0	2.1	ND	ND	ND
4	9.03	Undecane	C_11_H_24_	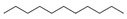	ND	ND	ND	ND	ND	ND	33.9	ND	ND	ND	ND	ND
5	9.40	2-Methyl-trans-decalin	C_11_H_20_	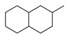	ND	ND	ND	ND	ND	ND	24.2	ND	ND	ND	ND	ND
6	9.60	2-Methyl-trans-decalin, isomer	C_11_H_20_	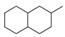	ND	ND	ND	ND	ND	ND	37.5	ND	ND	ND	ND	ND
7	9.61	2-Phenylethanol	C_8_H_10_O	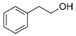	ND	ND	ND	ND	ND	ND	ND	1.2	ND	ND	ND	ND
8	9.69	2-Methyl-trans-decalin, isomer	C_11_H_20_	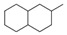	ND	ND	ND	ND	ND	ND	11.7	ND	ND	ND	ND	ND
9	9.89	2-Methyl-cis-decalin	C_11_H_20_	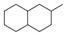	ND	ND	ND	ND	ND	ND	18.4	ND	ND	ND	ND	ND
10	9.98	2-Methyl-cis-decalin isomer	C_11_H_20_	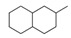	ND	ND	ND	ND	ND	ND	10.4	ND	ND	ND	ND	ND
11	10.09	2,6-Dimethyldecalin	C_12_H_22_	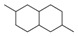	ND	ND	ND	ND	ND	ND	19.2	ND	ND	ND	ND	ND
12	10.27	Dodecane	C_12_H_26_	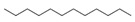	ND	ND	ND	ND	ND	ND	50.0	ND	ND	ND	ND	ND
13	10.70	Coumaran	C_8_H_8_O	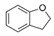	ND	ND	ND	ND	ND	ND	90.0	ND	ND	ND	ND	ND
14	10.75	2,4-Dimethylbenzaldehyde	C_9_H_10_O	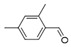	ND	ND	ND	ND	ND	ND	155.4	1.2	ND	ND	ND	ND
15	10.81	Hexylcyclohexane	C_12_H_24_	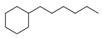	ND	ND	ND	ND	ND	ND	30.9	ND	ND	ND	ND	ND
16	10.90	m-Di-tert-butylbenzene	C_14_H_22_	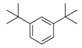	ND	ND	ND	ND	ND	ND	46.8	ND	ND	ND	ND	ND
17	11.39	1-Butoxy-1-isobutoxy-butane	C_12_H_26_O_2_	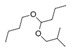	ND	ND	ND	2.7	ND	ND	ND	ND	ND	ND	ND	ND
18	11.65	1,1′-Bicyclohexyl	C_12_H_22_	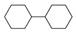	ND	ND	ND	ND	ND	ND	127.8	ND	ND	ND	ND	ND
19	11.88	3,4-Dimethylbenzamide	C_9_H_11_NO	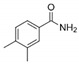	ND	ND	ND	ND	ND	ND	ND	ND	ND	1.2	ND	4.1
20	12.31	Tetradecane	C_14_H_30_	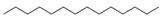	ND	ND	ND	ND	ND	ND	73.8	ND	ND	ND	ND	ND
21	13.05	2,6-Di-tert-butyl-P-benzoquinone	C_14_H_20_O_2_	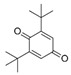	ND	ND	ND	ND	ND	ND	110.7	ND	ND	ND	ND	ND
22	13.39	2,4-Di-tert-butylphenol	C_14_H_22_O	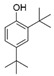	ND	1.5	ND	ND	ND	ND	1297.7	13.0	3.4	ND	ND	ND
23	13.60	Ethyl 4-ethoxybenzoate	C_11_H_14_O_3_	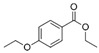	ND	ND	ND	ND	ND	ND	244.0	ND	ND	ND	ND	ND
24	14.49	3-Methyl-heptadecane	C_18_H_38_	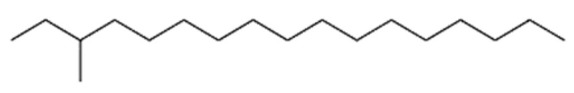	ND	ND	ND	ND	ND	ND	283.1	ND	ND	ND	ND	ND
25	14.81	Heptadecane	C_17_H_36_	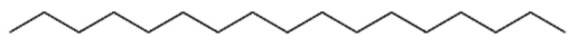	ND	ND	ND	ND	ND	ND	519.6	ND	ND	ND	ND	ND
26	15.07	6-Phenyldodecane	C_18_H_30_	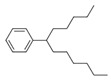	ND	ND	ND	ND	ND	ND	444.3	ND	ND	ND	ND	ND
27	15.21	4-Phenyldodecane	C_18_H_30_	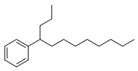	ND	ND	ND	ND	ND	ND	210.3	ND	ND	ND	ND	ND
28	15.39	Cyclo(prolyl-sarcosine)	C_8_H_12_N_2_O_2_	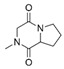	ND	ND	ND	ND	ND	ND	ND	33.8	ND	ND	ND	ND
29	15.50	DL-Alanyl-L-leucine	C_9_H_18_N_2_O_3_	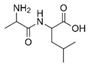	ND	ND	ND	ND	ND	ND	ND	22.4	3.1	ND	ND	ND
30	15.48	Cyclo(prolyl-sarcosine), isomer	C_8_H_12_N_2_O_2_	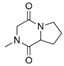	ND	7.4	ND	ND	ND	ND	ND	ND	ND	ND	ND	ND
31	15.70	2-Phenyldodecane	C_18_H_30_	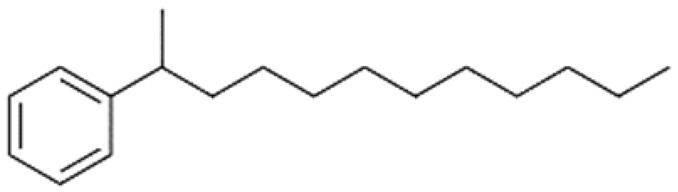	ND	ND	ND	ND	ND	ND	877.9	ND	ND	ND	ND	ND
32	15.76	n-Butylbenzenesulfonamide	C_10_H_15_NO_2_S	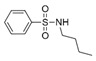	ND	ND	ND	ND	ND	ND	558.2	ND	ND	ND	ND	ND
33	15.84	5-Phenyltridecane	C_19_H_32_	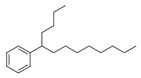	ND	ND	ND	ND	ND	ND	138.5	ND	ND	ND	ND	ND
34	16.04	Cyclo(L-prolyl-L-valine)	C_10_H_16_N_2_O_2_	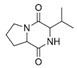	2.0	139.5	ND	ND	ND	ND	1653.6	405.9	2.6	ND	ND	2.9
35	16.31	Cyclo(L-prolyl-L-valine), isomer	C_10_H_16_N_2_O_2_	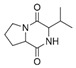	ND	ND	ND	ND	ND	ND	ND	80.8	ND	ND	ND	ND
36	16.45	Methyl palmitate (C16:0)	C_17_H_34_O_2_	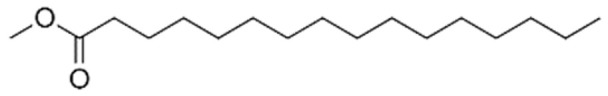	ND	ND	ND	ND	ND	ND	ND	ND	ND	1.0	ND	4.1
37	16.47	7,9-Di-t-butyl-1-oxaspiro(4,5)deca-6,9-diene-2,8-dione	C_17_H_24_O_3_	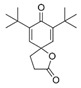	ND	ND	ND	ND	ND	ND	748.9	ND	ND	ND	ND	ND
38	16.56	Cyclo(prolyl-leucine)	C_11_H_18_N_2_O_2_	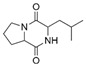	4.4	5.7	ND	ND	ND	ND	ND	38.3	ND	ND	ND	ND
39	16.80	Cyclo(prolyl-leucine), isomer	C_11_H_18_N_2_O_2_	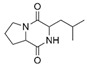	0.9	525.4	ND	ND	ND	ND	ND	600.8	1.5	ND	ND	1.6
40	16.91	Cyclo(prolyl-leucine), isomer	C_11_H_18_N_2_O_2_	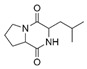	1.9	801.2	ND	ND	ND	ND	ND	667.5	2.2	ND	ND	1.0
41	17.02	Cyclo(prolyl-leucine), isomer	C_11_H_18_N_2_O_2_	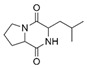	ND	115.2	ND	ND	ND	ND	ND	390.2	ND	ND	ND	0.5
42	17.19	Norhamane, N-EtAcyl	C_13_H_10_N_2_O	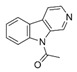	3.0	ND	ND	ND	ND	ND	tr	ND	ND	ND	ND	ND
43	17.25	2,4,6-Triisopropylphenol	C_14_H_20_O_2_	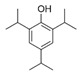	ND	ND	ND	ND	ND	ND	603.4	ND	ND	ND	ND	ND
44	17.74	Methyl iso-stearate (iC18:0)	C_19_H_38_O_2_	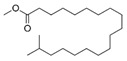	ND	ND	ND	ND	ND	ND	ND	ND	ND	1.0	ND	3.4
45	18.11	9,17-Octadecadienal	C_18_H_32_O	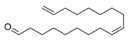	ND	ND	ND	ND	ND	ND	15280.5	ND	ND	ND	ND	ND
46	18.40	Cyclo(alanyl-phenylalanine)	C_12_H_14_N_2_O_2_	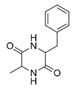	ND	ND	ND	ND	ND	ND	ND	23.3	ND	ND	ND	ND
47	18.40	DL-Alanyl-L-phenylalanine	C_12_H_16_N_2_O_2_	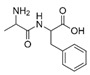	ND	ND	ND	ND	ND	ND	ND	ND	3.9	ND	ND	ND
48	18.92	Tert-octyldephenylamine	C_20_H_27_N	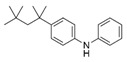	ND	ND	ND	ND	ND	ND	ND	9.6	ND	ND	ND	ND
49	18.97	Cyclo(phenylalanyl-valine)	C_14_H_18_N_2_O_2_	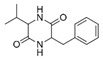	ND	ND	ND	ND	ND	ND	459.6	55.1	ND	ND	ND	ND
50	19.06	Octinoxate	C_18_H_26_O_3_	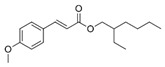	ND	ND	ND	ND	ND	ND	502.6	ND	ND	ND	ND	ND
51	19.07	Cyclo(phenylalanyl-valine), isomer	C_14_H_18_N_2_O_2_	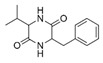	ND	6.1	ND	ND	ND	ND	ND	ND	ND	ND	ND	ND
52	19.30	Dioctyl adipate	C_22_H_42_O_4_	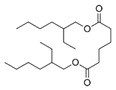	ND	171.2	ND	ND	ND	ND	409.5	109.7	ND	ND	ND	ND
53	19.45	2,2′-Methylenebis(6-tert-butyl-p-cresol)	C_23_H_32_O_2_	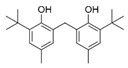	ND	ND	ND	ND	ND	ND	tr	ND	tr	ND	ND	3.2
54	19.49	Cyclo(prolyl-phenylalanine)	C_14_H_16_N_2_O_2_	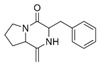	5.8	tr	ND	ND	ND	ND	284.1	224.2	ND	ND	ND	ND
55	19.51	2,2′-Methylenebis(6-tert-butyl-p-cresol), isomer	C_23_H_32_O_2_	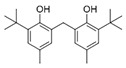	ND	ND	ND	ND	ND	ND	ND	ND	ND	ND	ND	5.0
56	19.61	2,2′-Methylenebis(6-tert-butyl-p-cresol), isomer	C_23_H_32_O_2_	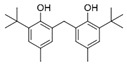	ND	ND	ND	ND	ND	ND	ND	ND	2.1	ND	ND	431.7
57	19.78	Cyclo(prolyl-phenylalanine), isomer	C_14_H_16_N_2_O_2_	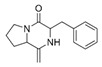	ND	ND	ND	ND	ND	ND	529.6	406.0	ND	ND	ND	ND
58	20.25	2,2′-Methylenebis(6-tert-butyl-p-cresol), isomer	C_23_H_32_O_2_	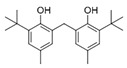	ND	ND	406.6	328.8	39.1	4.9	ND	ND	ND	ND	ND	ND
59	20.35	Di-2-propylpentyl-phthalate	C_24_H_38_O_4_	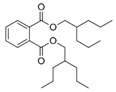	ND	ND	ND	ND	ND	ND	468.0	ND	ND	ND	ND	ND
60	21.19	2-Ethoxy-2′-ethyloxanilide	C_18_H_20_N_2_O_3_	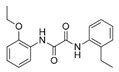	5.8	ND	ND	ND	ND	ND	ND	ND	ND	ND	ND	ND
61	22.31	Di-2-ethylhexyl-isophthalate	C_24_H_38_O_4_	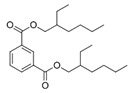	ND	ND	ND	ND	ND	ND	89.7	ND	ND	ND	ND	ND
62	22.91	Erucamide	C_22_H_43_NO	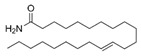	ND	ND	ND	ND	ND	ND	165.3	ND	ND	ND	ND	ND
63	23.03	Squalene	C_30_H_50_	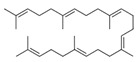	ND	ND	ND	ND	ND	ND	168.7	ND	ND	ND	ND	ND
64	24.01	Cyclo(phenylalanyl-phenylalanine)	C_18_H_18_N_2_O_2_	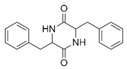	ND	ND	ND	ND	ND	ND	ND	10.7	ND	ND	ND	ND

tr indicates integration inferior to 0.1 × 10^6^; “ND”: not detected.

**Table 4:** GC-MS analysis (area ×10^6^) with derivatization for BC extracts of *B. megaterium* culture and CS2 extracts of control medium.

N°	RT (min)	Compound	Formula	Structure	CS2						BC					
					Cyclo	Dichlo	EtAc	BuOH	Water	Raw	Cyclo	Dichlo	EtAc	BuOH	Water	Raw
1′	6.87	Carbonic acid	C_1_H_2_O_3_	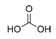	ND	ND	ND	ND	1.4	ND	1.4	ND	ND	ND	ND	ND
2′	7.11	Ethylene Glycol	C_2_H_6_O_2_	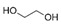	ND	ND	ND	ND	ND	ND	ND	ND	ND	0.4	ND	ND
3′	7.22	Pyruvic acid	C_3_H_4_O_3_	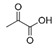	ND	1.5	3.4	2.0	1.9	0.4	3.9	2.8	3.0	ND	ND	ND
4′	7.28	Propylene glycol	C_3_H_8_O_2_	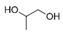	ND	ND	ND	ND	ND	ND	ND	ND	1.8	1.0	0.3	ND
5′	7.81	2,3-Butanediol	C_4_H_10_O_2_	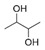	ND	ND	ND	ND	ND	ND	5.6	586.0	981.3	1511.9	60.2	1.85
6′	7.97	2,3-Butanediol, isomer	C_4_H_10_O_2_	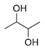	ND	ND	ND	ND	ND	ND	ND	2.2	1.9	1.8	ND	ND
7′	8.33	Lactic Acid	C_3_H_6_O_3_	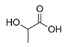	ND	ND	3.2	ND	ND	ND	2.1	2.5	5.2	47.9	36.0	5.28
8′	8.45	2-Hydroperoxytetrafuran	C_4_H_8_O_3_	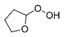	ND	6.9	27.2	ND	2.4	0.2	50.7	50.6	36.6	ND	ND	ND
9′	8.62	Glycolic acid	C_2_H_4_O_3_	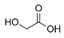	ND	ND	ND	ND	ND	ND	ND	ND	0.3	0.3	0.6	ND
10′	8.94	Alanine	C_3_H_7_O_2_	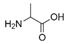	ND	ND	ND	ND	ND	ND	ND	ND	ND	ND	2.6	ND
11′	8.98	2-Propyl-1-pentanol	C_8_H_18_O_1_	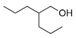	ND	ND	ND	1.5	ND	tr	ND	ND	ND	0.2	ND	ND
12′	10.29	L-Norvaline	C_5_H_11_O_2_N_2_	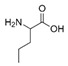	ND	ND	ND	ND	ND	ND	ND	ND	ND	ND	2.6	ND
13′	10.87	Glycerol	C_3_H_8_O_3_	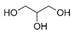	ND	ND	2.5	1.9	ND	ND	1.7	ND	0.3	ND	ND	ND
14′	10.94	Phosphoric acid	PO_4_H_3_	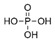	ND	ND	1.2	1.4	1.5	ND	1.9	0.5	0.3	0.9	42.1	ND
15′	11.13	L-Isoleucine	C_6_H_13_O_6_N_2_	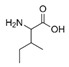	ND	ND	ND	ND	ND	ND	ND	ND	ND	ND	1.8	ND
16′	11.45	Glyceric acid	C_3_H_6_O_4_	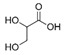	ND	1.2	5.8	0.5	ND	ND	ND	ND	0.3	0.6	0.8	ND
17′	11.68	Uracil	C_4_H_4_O_2_N_2_	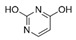	ND	ND	ND	ND	ND	ND	ND	ND	1.2	0.6	ND	ND
18′	11.73	Serine	C_3_H_7_O_3_N_3_	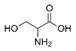	ND	ND	ND	ND	ND	ND	ND	ND	ND	ND	0.4	ND
19′	11.80	1-Monoacetin	C_5_H_10_O_4_	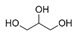	ND	3.6	2.5	ND	ND	ND	ND	ND	ND	ND	ND	ND
20′	11.96	L-Threonine	C_4_H_9_O_3_N_3_	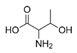	ND	ND	ND	ND	ND	ND	ND	ND	ND	ND	0.5	ND
21′	12.94	L-Threitol	C_4_H_10_O_4_	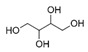	ND	ND	ND	ND	ND	ND	ND	ND	ND	5.7	1.0	ND
22′	14.15	Phenylalanine	C_9_H_11_O_2_N_2_	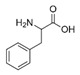	ND	ND	ND	ND	ND	ND	ND	ND	ND	ND	0.5	ND
23′	14.85	Tridecanoic acid	C_13_H_26_O_2_	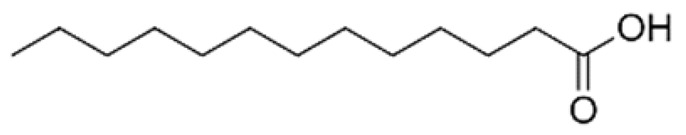	ND	ND	ND	ND	ND	ND	0.8	ND	ND	ND	ND	ND
24′	15.07	Ribonic acid	C_5_H_10_O_6_	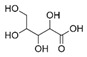	ND	ND	ND	ND	ND	ND	ND	ND	ND	0.6	ND	ND
25′	15.15	Allofuranose	C_6_H_12_O_6_	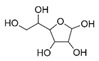	ND	ND	4.0	ND	ND	ND	ND	ND	ND	ND	ND	ND
26′	15.22	Sorbofuranose	C_6_H_12_O_6_	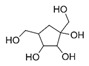	ND	0.8	38.8	97.6	ND	5.2	ND	ND	ND	ND	ND	ND
27′	15.30	Fructofuranose	C_6_H_12_O_6_	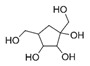	ND	3.5	205.2	413.5	58.3	22.2	ND	ND	ND	ND	ND	ND
28′	15.36	Fructopyranose	C_6_H_12_O_6_	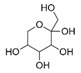	ND	ND	19.7	27.9	1.3	2.4	ND	ND	ND	ND	ND	ND
29′	15.40	D-Pinitol	C_7_H_14_O_6_	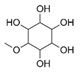	ND	ND	ND	ND	ND	ND	ND	ND	ND	ND	1.0	ND
30′	15.43	Arabinopyranose	C_5_H_10_O_5_	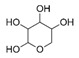	ND	ND	54.2	8.3	1.3	ND	ND	ND	ND	ND	ND	ND
31′	15.52	Talofuranose	C_6_H_12_O_6_	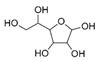	ND	ND	7.2	72.4	5.4	2.3	ND	ND	ND	ND	ND	ND
32′	15.57	Myristic acid	C_14_H_28_O_2_	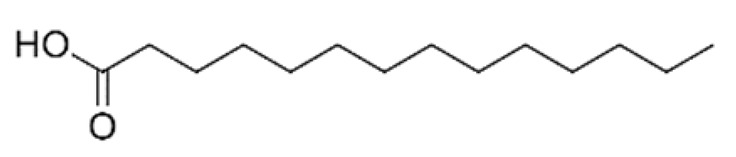	ND	ND	ND	ND	ND	ND	3.1	ND	ND	ND	ND	ND
33′	15.63	Lyxopyranose	C_5_H_10_O_5_	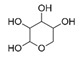	ND	16.0	866.3	349.7	229.7	52.5	ND	ND	ND	0.6	1.4	ND
34′	15.65	Myristoleic acid	C_14_H_26_O_2_	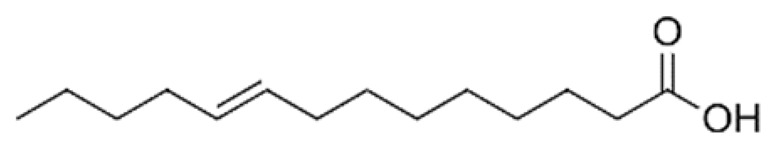	ND	ND	ND	ND	ND	ND	7.2	ND	ND	ND	ND	ND
35′	15.89	Mannopyranose	C_6_H_12_O_6_	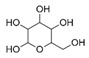	ND	ND	9.5	999.0	219.0	146.5	ND	ND	ND	3.2	ND	ND
36′	16.01	Talopyranose	C_6_H_12_O_6_	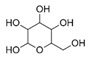	ND	ND	101.1	11.7	7.6	6.5	ND	ND	ND	ND	ND	ND
37′	16.07	Allopyranose	C_6_H_12_O_6_	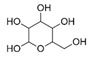	ND	ND	169.3	156.5	42.7	47.1	ND	ND	ND	ND	ND	ND
38′	16.09	D-Galactose oxime	C_6_H_13_O_6_N_6_	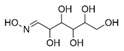	ND	ND	ND	ND	ND	ND	ND	ND	ND	ND	0.3	ND
39′	16.31	Pentadecanoic acid	C_15_H_30_O_2_	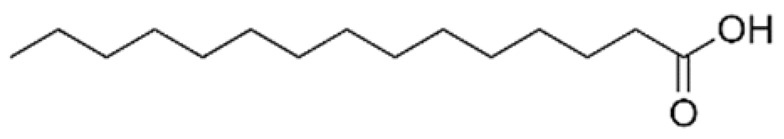	ND	ND	ND	ND	ND	ND	153.9	1.3	ND	ND	ND	ND
40′	16.43	Glucopyranose	C_6_H_12_O_6_	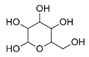	ND	2.2	7.9	2393.7	49.6	15.7	ND	ND	ND	0.7	1.4	ND
41′	16.56	Ribonic acid, isomer	C_5_H_10_O_6_	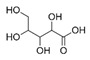	ND	ND	ND	ND	ND	ND	ND	ND	ND	ND	0.5	ND
42′	17.15	Palmitic acid	C_16_H_32_O_2_	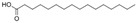	ND	ND	ND	ND	ND	ND	185.9	4.3	ND	ND	ND	ND
43′	17.16	Myo-Inositol	C_6_H_12_O_6_	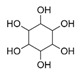	ND	ND	ND	ND	ND	ND	ND	ND	ND	ND	0.5	ND
44′	17.58	Margaric acid	C_17_H_34_O_2_	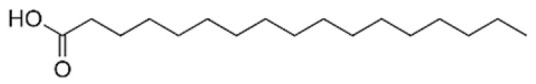	ND	ND	ND	ND	ND	ND	6.8	0.4	ND	ND	ND	ND
45′	17.63	Psicofuranose	C_6_H_12_O_6_	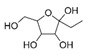	ND	ND	ND	2.4	ND	ND	ND	ND	ND	0.9	ND	ND
46′	18.24	Oleic acid	C_18_H_34_O_2_	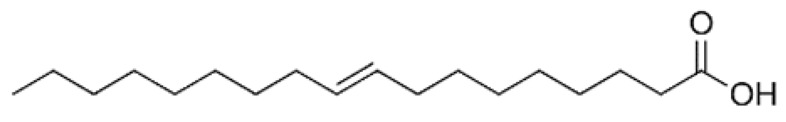	ND	ND	ND	ND	ND	ND	1025.0	45.5	ND	ND	ND	ND
47′	18.35	Stearic acid	C_18_H_36_O_2_	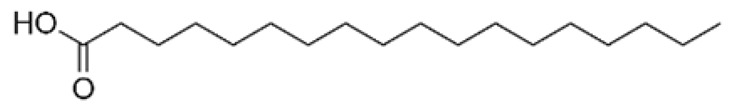	ND	ND	ND	ND	ND	ND	19.2	1.0	ND	ND	ND	ND
48′	20.37	Turanose	C_12_H_22_O_11_	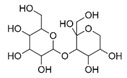	ND	ND	ND	3.6	ND	ND	ND	ND	ND	ND	ND	ND

tr indicates integration inferior to 0.1 × 10^6^; “ND”: not detected.

## Data Availability

Data are contained within the article.
